# Delayed conversion from central venous catheter to non‐catheter hemodialysis access associates with an increased risk of death: A retrospective cohort study based on data from a large dialysis provider

**DOI:** 10.1111/hdi.12831

**Published:** 2020-03-05

**Authors:** Jochen G. Raimann, Fang‐I Chu, Sean Kalloo, Hanjie Zhang, Frank Maddux, Yuedong Wang, Peter Kotanko

**Affiliations:** ^1^ Research Division Renal Research Institute New York New York USA; ^2^ Department Radiation Oncology University of California‐Los Angeles Los Angeles California USA; ^3^ Department of Statistics & Applied Probability University of California‐Santa Barbara Santa Barbara California USA; ^4^ Division of Nephrology Columbia University College of Physicians and Surgeons New York New York USA; ^5^ Global Medical Office Fresenius Medical Care North America Waltham Massachusetts USA; ^6^ Department of Nephrology Icahn School of Medicine at Mount Sinai Health System New York New York USA

**Keywords:** dialysis initiation, access conversion, arteriovenous fistula, arteriovenous graft, central venous catheter, predialysis care

## Abstract

**Background:**

Hemodialysis initiation using a central venous catheter (CVC) poses an increased risk of death. Conversion to an arterio‐venous graft or fistula (AVF, AVG) improves outcomes. The relationship of primary dialysis access and timing of conversion from CVC to either AVF or AVG to all‐cause mortality was investigated.

**Methods:**

Two retrospective analyses in incident hemodialysis patients commencing treatment from January 2010 to December 2014 in dialysis clinics in the United States were conducted. *Analysis 1* stratified as per access at initiation and those commencing with CVC were further stratified into (a) those that had a CVC, AVF, or AVG the entire year; (b) those that were converted to either AVF or AVG within either (i) the first or (ii) the second 6 months. Kaplan Meier analysis and Cox regression analysis were employed. *Analysis 2* included all CVC patients investigating the relationship between access conversion time and mortality risk using a Cox proportional hazards model depicting the hazard ratio (HR) as a spline function over time.

**Results:**

Two subsets from initial 78,871 patients were studied. In *Analysis 1* both AVF (referent) and AVG [HR 1.12 (0.97 to 1.30)] associated with a better outcome than CVC [HR 1.55 (1.38 to 1.74)] during follow‐up. Lower mortality risk was seen for early switch from a CVC to AV access within the first 6 months [HR = 1.04 (0.97–1.13)] compared to a later switch [HR = 1.23 (1.10–1.38)]. *Analysis 2* indicated that a CVC to AVF switch resulted in improved survival. *Analysis* 2 indicated early conversion to confer a survival benefit for CVC to AVG switch.

**Discussion and Conclusion:**

AVF and AVG show a survival benefit over CVC. Early conversion from CVC to either access improves survival. This emphasizes the importance of early preparation for dialysis by creation of an AVF or AVG and to convert CVCs early.

## Introduction

Hemodialysis can be started using one of three main types of vascular accesses: arteriovenous fistulas (AVFs), arteriovenous grafts (AVGs), and tunneled cuffed CVC. The superiority of AVF and AVG over CVC in terms of survival, hospitalizations, and other outcomes has been well established over the last decades^1^, ^2^, ^3^, ^4^; however, still 80.3% of all patients commenced hemodialysis with a CVC in 2014.^5^ Change of access from CVC to either AVF or AVG have been reported to associate with a favorable change in markers of nutrition, inflammation, anemia,^4^ hospitalization,^6^, ^7^ and mortality,^1^, ^8^, ^9^, ^10^, ^11^ but still 68.3% of hemodialysis patients were using a CVC as their primary access.^5^ Data also suggested that not only the conversion per se from CVC to either AVF or AVG matters but also timing of conversion plays a role,^1^, ^8^ a finding that also held true in a large international, elderly population.^10^

Given the importance and the substantial positive effects possibly achievable by (early) conversion from CVC to either AVF or AVG, we focused in this analysis on mortality risk as a function of time following hemodialysis initiation. This analysis tests the hypothesis that early conversion for those commencing renal replacement therapy using a CVC will show a higher survival probability as compared to that when starting hemodialysis with a CVC and remaining on it for a longer time.

## METHODS

This is a retrospective, longitudinal cohort study in incident hemodialysis patients with complete data commencing renal replacement therapy in the 2368 clinics of Fresenius Medical Care dialysis clinics in the United States between January 01, 2010 and December 31, 2014. The study consisted of two separate analyses with one stratifying dialysis access and conversion during the first year and following patients' outcomes over a 4‐year period and the other one quantifying the risk of death as a function of time of conversion from catheter to either AVF or AVG throughout the entire study.

### Study design

The study consisted of two separate analyses (1) *Analysis 1* investigated different access types at dialysis initiation and the respective switches from CVC to either AVF or AVG in patients who survived the first year of the study and the measures of the first year were used to establish a baseline and the following 4 years served as the follow‐up period. All patients were stratified into those commencing HD with either an AVF, AVG, or CVC and remaining on the same access for the entire first year, and those CVC patients switching to AVF or AVG either within the first or the second half of the first year of HD. (2) *Analysis 2* modeled the risk of death for those that switched to either AVF or AVG in a continuous fashion as a spline function over time relative to those remaining on CVC as the primary dialysis access. Patients who were transferred to other clinics, switched modality, and received a transplant were censored. While the source population of both analyses is the same, *Analysis 2* only included those that started on a CVC (and either switched or remained on a CVC) regardless of outcome in the first year (*Analysis 1* only included survivors of Year 1).

An Institutional Review Board has waived the need for review and determined this study as exempt under the provisions of 45 CFR 46 Section 101(b) as per the United States Health Insurance Portability and Accountability Act. Given the nature of this retrospective study determined as exempt, no informed consent from studied patients had been obtained. Manuscript development followed the recommendations of the STROBE guidelines.^12^

### Patient population

**Figure 1:**
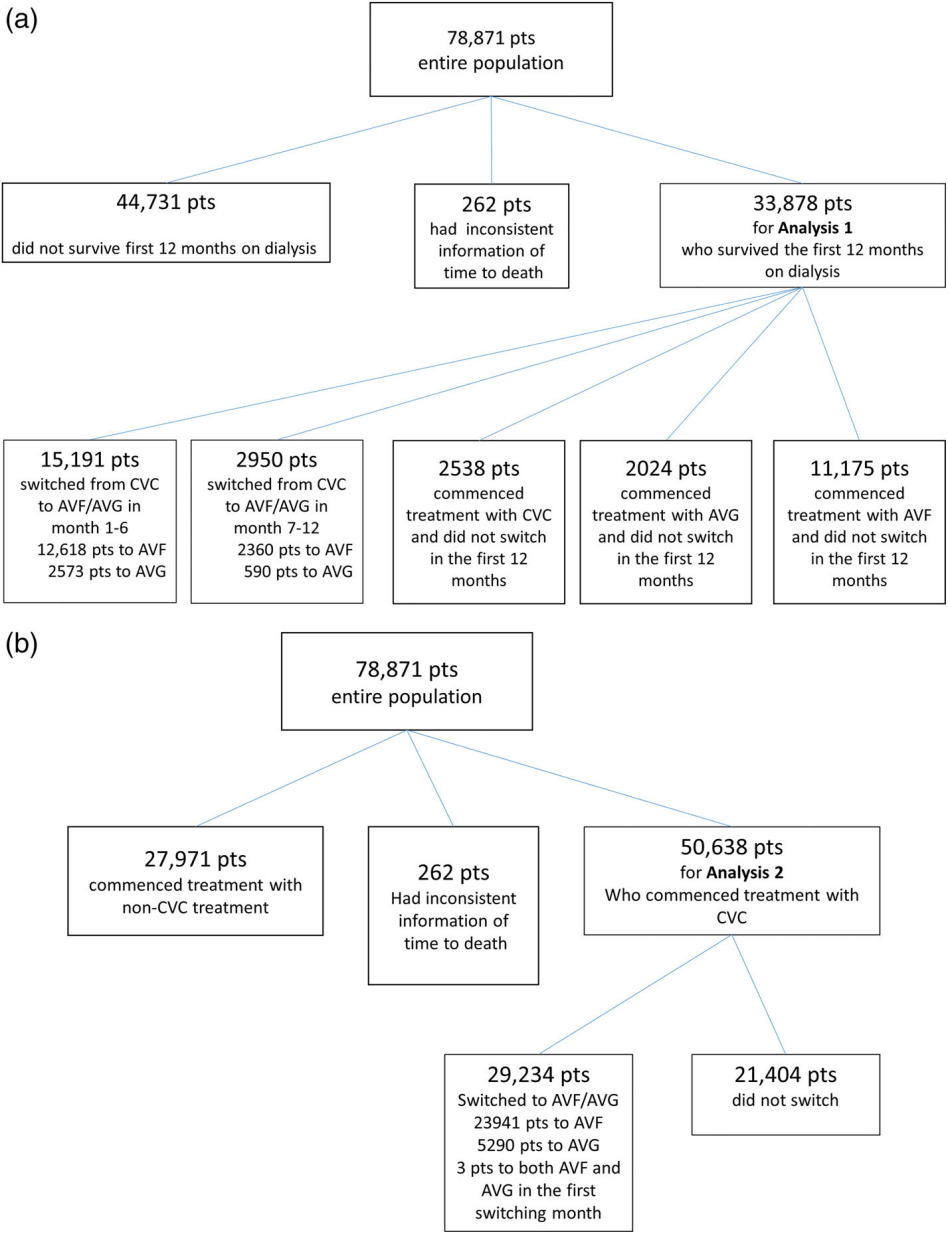
Study flowchart. [Color figure can be viewed at wileyonlinelibrary.com]

An entire cohort of population 78,871 patients was studied. The study population of *Analysis 1* and *Analysis 2* was subsetted from the entire cohort according to respective inclusion criteria. Figure 1a,b shows detailed flowcharts of the study population for both analyses.

**Figure 2:**
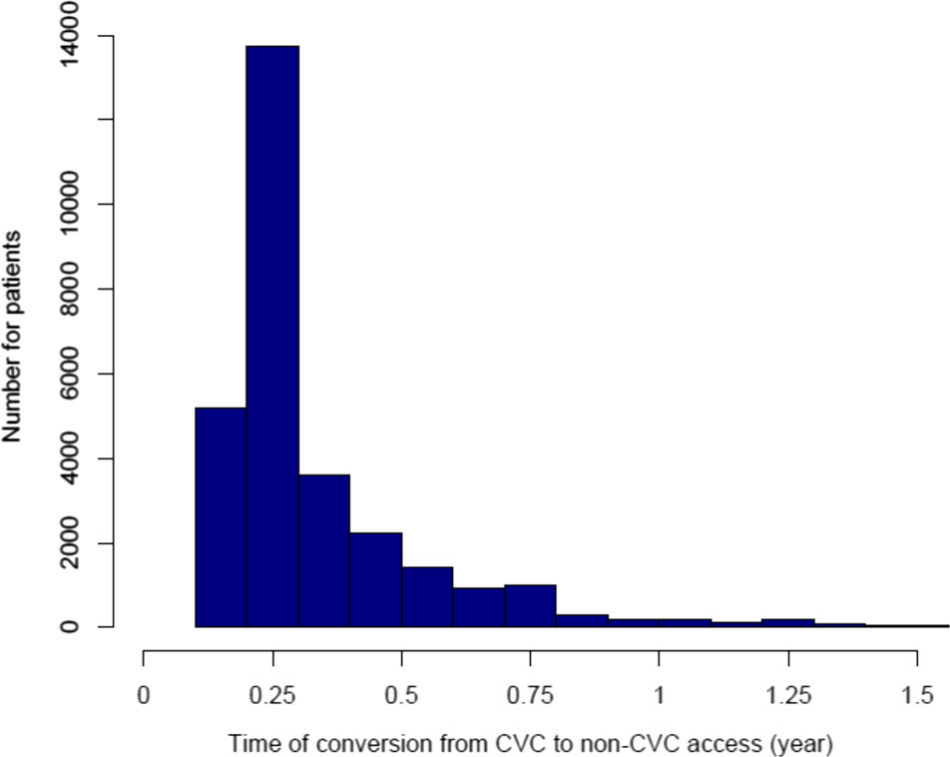
Histogram of times using central venous catheter (CVC) until conversion to either arteriovenous fistula or graft (non‐CVC). [Color figure can be viewed at wileyonlinelibrary.com]

*Analysis 1* studied 33,878 patients who commenced maintenance hemodialysis treatment with either CVC, AVF, or AVG as the primary vascular access at facilities operated by a large dialysis provider between January 2010 and December 2014 and survived the first 12 months. In patients commencing treatment using a CVC most patients were converted to either AVG or AVF within the first year. Significant differences between the groups are explained by the large sample size and the magnitude of the differences does not suggest contrasts in demographic and clinical parameters that are clinically relevant (Table 1). *Analysis 2* studied 50,638 patients where 29,234 (57.7%) were converted during the study period (Table 2). *Analysis 1* includes patients who commenced with CVC, AVF, or AVG and survived/not censored in the first year, while analysis 2 includes all patients who commenced with CVC. Therefore, the numbers of patients for these two analyses are different and there is a large number of patients in Analysis 2 who did not switch and died in the first year. Figure 2 shows the distribution of switch times in the population of Analysis 2.

**Table 1:** Descriptive statistics for patients who stayed with CVC, AVF, or AVG and CVC patients that switched either during the first or the second half of the first year to a non‐CVC access. Cell contents are mean (SD) or percentages for categorical variables

	AVF	AVG	CVC (entire 12 months)	Difference between AVF, AVG, and the CVC at all times group	CVC (switched months 1 to 6)	CVC (switched months 7 to 12)	Difference between groups of CVC switch during months 1 to 6 compared to the group months 7 to 12.
N [count]	11175	2024	2538		15191	2950	
Age [years]	63.3 (14.0)	65.3 (13.6)	62.4 (16.0)	*p* < 0.001	62.4 (14.5)	61.0 (14.7)	*p* < 0.001
White race [%]	67.7	52.2	64.9	*p* < 0.001	65.0	60.0	*p* < 0.001
Male gender [%]	63.6	42.8	48.0	*p* < 0.001	56.6	52.4	*p* < 0.001
Diabetic [%]	63.4	69.1	63.2	*p* < 0.001	67.9	66.6	*p* = 0.19
Hispanic ethnicity [%]	14.6	11.1	13.1	*p* < 0.001	16.2	15.9	*p* = 0.73
Pre HD SBP [mmHg]	148.3 (16.2)	148.9 (16.4)	148.6 (19.1)	*p* = 0.39	148.7 (17.0)	149.3 (17.4)	*p* = 0.13
Pre HD DBP [mmHg]	76.1 (10.3)	74.9 (10.2)	78.8 (12.0)	*p* < 0.001	77.1 (10.7)	79.0 (11.3)	*p* < 0.001
Post HD SBP [mmHg]	139.3 (14.7)	140.0 (14.4)	142.4 (16.9)	*p* < 0.001	140.9 (15.3)	142.2 (15.5)	*p* < 0.001
Post HD DBP [mmHg]	72.5 (8.7)	71.3 (8.2)	75.9 (10.5)	*p* < 0.001	73.7 (9.0)	75.6 (9.5)	*p* < 0.001
IDWG [% body weight]	2.8 (1.0)	2.8 (1.0)	2.8 (1.0)	*p* = 0.25	2.9 (0.9)	2.9 (1.0)	*p* = 0.23
Ultrafiltration rate [mL/h/kg body weight]	7.6 (2.6)	7.8 (2.8)	7.7 (2.8)	*P <* 0.01	7.8 (2.6)	7.7 (2.6)	*p* = 0.26
EPO [unit]	3746.4 (3303.5)	4020.4 (3441.3)	5145.4 (4092.2)	*p* < 0.001	4470.2 (3554.6)	4856.4 (3751.0)	*p* < 0.001
Albumin [g/dL]	3.8 (0.3)	3.8 (0.3)	3.6 (0.4)	*p* < 0.001	3.8 (0.4)	3.7 (0.4)	*p* < 0.001
NLR	4.0 (2.3)	3.8 (2.1)	4.7 (2.9)	*p* < 0.001	4.2 (2.5)	4.2 (2.6)	*p* = 0.56
enPCR [g/kg/d]	0.9 (0.2)	0.9 (0.2)	0.8 (0.2)	*p* < 0.001	0.9 (0.2)	0.9 (0.2)	*p* < 0.001
eKt/V	1.6 (0.3)	1.6 (0.3)	1.5 (0.3)	*p* < 0.001	1.5 (0.3)	1.5 (0.3)	*p* < 0.001
BMI [kg/m^2^]	30.3 (10.2)	29.7 (12.0)	29.0 (15.9)	*p < 0.001*	30.1 (11.6)	30.1 (10.8)	*p = 0.84*

AVF = arterio‐venous fistula; AVG = arterio‐venous graft; BMI = body mass index.; CVC = central‐venous catheter; DBP = diastolic blood pressure; enPCR = equilibrated normalized protein catabolic rate; EPO = erythropoietin dose; HD = hemodialysis; IDWG = interdialytic weight gain; NLR = neutrophil‐lymphocyte ratio; SBP = systolic blood pressure.

**Table 2:** Descriptive statistics for all patients that commenced hemodialysis treatment with a CVC, those that are converted from CVC to a non‐CVC access (AVG or AVF) during the first year and those that are not

	All patients	Patients with access conversion	Patients with no access conversion
N [count]	50638	29234	21404
Age [years]	63.7 (15.0)	62.8 (14.6)	64.9 (15.5)
White race [per %]	71.1	67.4	76.2
Male gender [per %]	56.1	56.8	55.2
Diabetic [per %]	62.4	66.4	56.9
Hispanic ethnicity [per %]	12.9	14.6	10.6
Pre HD SBP [mmHg]	142.1 (23.3)	145.1 (22.3)	138.0 (24.1)
Pre HD DBP [mmHg]	75.1 (13.7)	76.2 (13.3)	73.5 (14.0)
Post HD SBP [mmHg]	143.7 (22.3)	146.8 (21.3)	139.5 (22.9)
Post HD DBP [mmHg]	75.6 (12.8)	76.8 (12.4)	73.8 (13.2)
IDWG [% body weight]	2.2 (1.3)	2.1 (1.3)	2.2 (1.4)
Ultrafiltration rate [mL/h/kg body weight]	6.4 (3.1)	6.4 (3.0)	6.4 (3.3)
EPO [unit]	4750.2 (5141.9)	4954.9 (5180.3)	4470.8 (5075.7)
Albumin [g/dL]	3.4 (0.5)	3.4 (0.5)	3.3 (0.6)
NLR	6.9 (6.6)	6.3 (5.8)	7.7 (7.6)
enPCR [g/kg/d]	0.7 (0.2)	0.7 (0.2)	0.7 (0.2)
eKt/V	1.4 (0.4)	1.4 (0.4)	1.4 (0.5)
BMI [kg/m^2^]	30.5 (13.1)	30.6 (12.7)	30.3 (13.6)

AVF = arterio‐venous fistula; AVG = arterio‐venous graft; BMI = body mass index.; CVC = central‐venous catheter; DBP = diastolic blood pressure; enPCR = equilibrated normalized protein catabolic rate; EPO = erythropoietin dose; HD = hemodialysis; IDWG = interdialytic weight gain; NLR = neutrophil‐lymphocyte ratio; SBP = systolic blood pressure.

### Measurements

Information on dialysis access, demographic (age, white race, gender, diabetes, Hispanic ethnicity) and dialysis treatment‐related data (were extracted from the medical records). Data of laboratory results were taken from routine laboratory assessments in the central database that all were performed in two centralized laboratories by trained and certified personnel.

### Sensitivity analyses

Switch to AVF or AVG is defined as the first time when AVF or AVG was used after initial use of CVC. Some patients switched between CVC, AVF, and AVG multiple times. Sensitivity analyses excluding those who switched multiple times from CVC to AVF or AVG were conducted.

### Statistical analyses

Descriptive statistics for demographic and baseline characteristics are summarized as mean ± SD for continuous variables percentages and 95% confidence intervals for categorical parameters and point estimates with 95% confidence intervals for probabilistic models, as appropriate. Comparison between groups were conducted using t test and one‐way ANOVA for continuous variables and Chi‐squared test for categorical variables. A two‐sided p‐value<0.05 was considered statistically significant. Analyses were done in R version 3.0.2 (“Frisbee Sailing”; R Foundation for Statistical Computing; Vienna, Austria)^13^ using packages *survival* and *splines*.

### Survival analysis

*Analysis 1* consists of Kaplan Meier survival curves and employed log‐rank test for comparison between all groups (i.e., those either commencing HD with either an AVF, AVG, or CVC and remaining on the same access for the entire year, and those switching from CVC to AVF or AVG either within the first or the second half of the first year of HD). Further Cox proportional hazards (Cox PH) models were constructed to quantify the risk of death after adjustment for various relevant factors.

For *Analysis 2* Cox PH models for switch from CVC to AVF and AVG, respectively, were constructed and spline functions fitted along a time axis depicting the hazard ratio of death after hemodialysis initiation over the entire study duration with those remaining for the entire time on a CVC as the reference group. The result from Cox PH models with spline function replaced by a step function of every 2 months were obtained as validation.

## RESULTS

### Survival analysis

**Figure 3:**
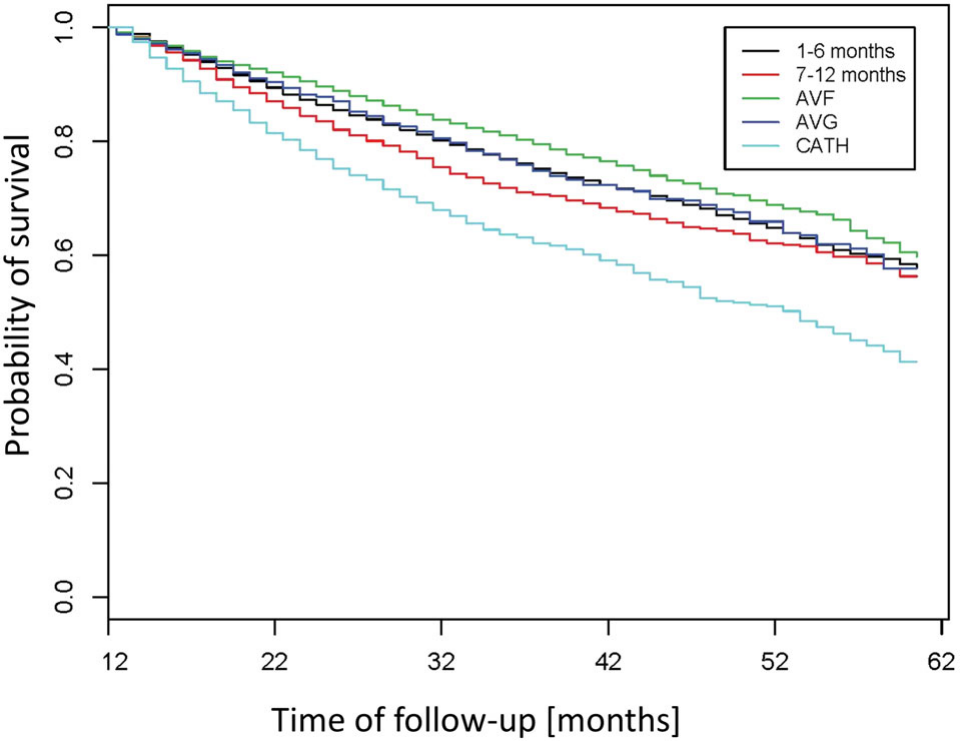
Kaplan–Meier survival curves depicting survival probability of those as a function of time over the follow‐up period after the first year of dialysis. All patients were stratified into those commencing hemodialysis with either arteriovenous fistula (AVF) or graft (AVG) or central venous catheter (CVC) and remaining on the same access for the entire year, and those switching from CVC to either AVF or AVG either within the first or the second half of the first year of hemodialysis. [Color figure can be viewed at wileyonlinelibrary.com]

**Figure 4:**
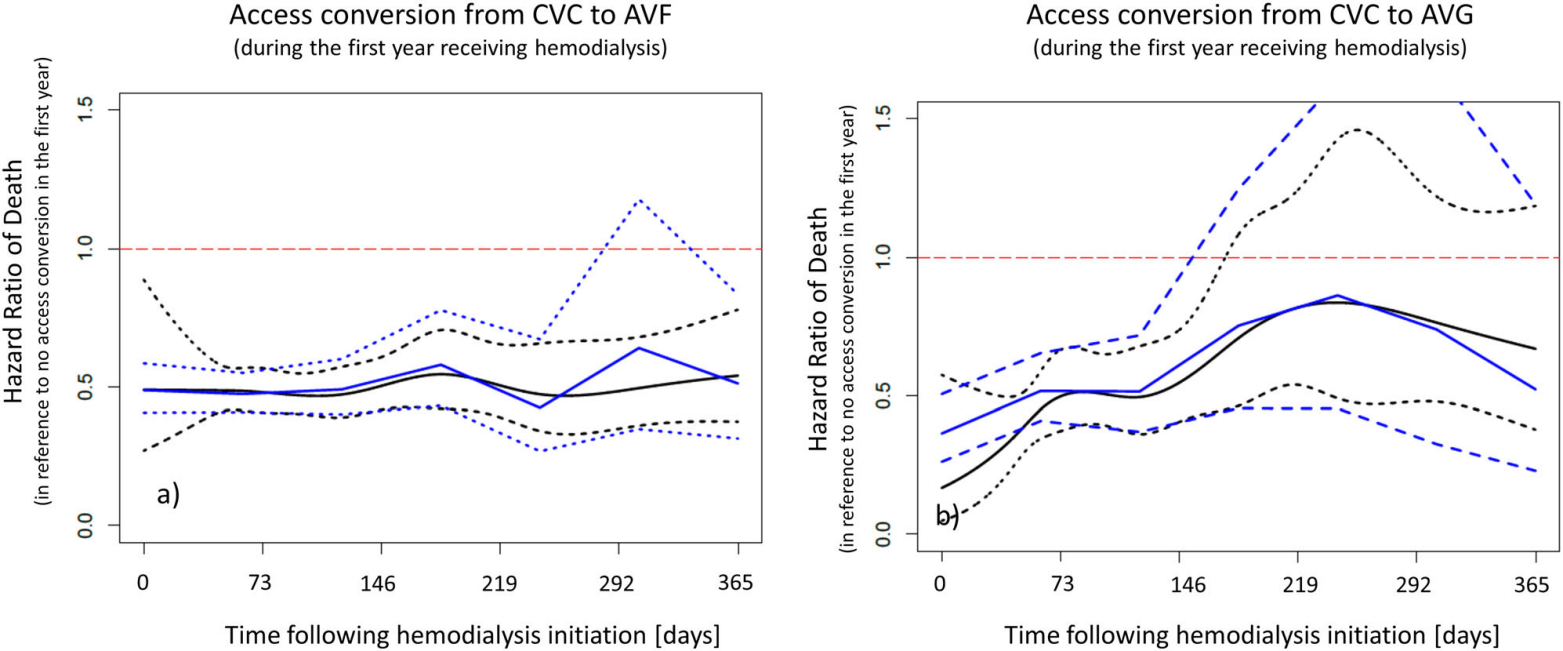
Spline function depicting hazard ratio of death from all causes in the year following hemodialysis initiation in patients that commenced treatment using a central‐venous catheter (CVC) and were converted from to either arteriovenous fistula (a: AVF) or graft (b: AVG). Solid black lines are spline estimates and solid blue lines are estimated step function of every two months. Dashed lines are 95% confidence intervals. [Color figure can be viewed at wileyonlinelibrary.com]

Kaplan Meier curves show a clear survival advantage for those commencing treatment using AVF (Figure 3), compared to those commencing using CVC that either switched or did not. Furthermore, the curves suggest that early conversion (<6 months following HD initiation) from CVC to either AVF or AVG (Figure 3, Figure S1, and Table S1) associates with a significantly improved survival probability. Of note, based on the log‐rank tests all p‐values for pairwise comparisons were smaller than 0.001 except for the comparison AVG versus conversion in the first 6 months with a p‐value 0.76. In a multivariable Cox Proportional Hazards model these relationships remained consistent with the lowest risk for those commencing with AVF (referent), followed by those commencing and remaining on AVG [hazard ratio (HR) 1.12 (95% CI 0.97 to 1.30)] and those starting and remaining on CVC showing the highest risk of death for those [HR 1.55 (95% CI 1.38 to 1.74)]. Of note, those patients who started with CVC and switched within the first 6 months of treatment had a risk of death comparable to those starting and remaining on AVF or AVG [1.04 (95% CI 0.97 to 1.13)], whereas those with access conversion in the second half of the first year had a significantly higher risk [1.23 (95% CI 1.10 to 1.38)]. It is of note that the associations found in the univariate analyses remained consistent after adjustment for all covariates included in the Cox Proportional Hazards model (Table 3, Table S2). Consistent with *Analysis 1*, *Analysis 2* indicated that a CVC‐to‐AVF switch resulted in a significantly lower HR of all‐cause mortality throughout the entire first year following dialysis initiation compared to remaining on CVC (black solid curve as spline estimates and dashed curve as 95% CI in Figure 4a). This observation aligns with the obtained HR of the Cox PH model with step function of every 2 months of switching time (blue solid curve as estimates of step function and dashed curve as 95% CI in Figure 4a). To some extent in contrast, a CVC‐to‐AVG switch also seems to improve survival but this improvement only showed to be significantly different at the 5% significance level during the first half of the first year. Similarly, this observation aligns with the obtained HR of the Cox PH model with step function (Figure 4b). However, it is of note that a particularly accentuated increase in survival benefit is found with access conversion during the first 6 months on dialysis, after which the upper bound of the 95% confidence interval crossed the HR of 1 (indicating a lack of significant difference; Figure 4a and Figure 4b).

**Table 3:** Hazard ratio of death estimates from Cox regression analysis for 22,146 patients who either stayed with CVC, AVF, or AVG and CVC patients that switched either during the first or the second half of the first year to a non‐CVC access. Only access related HRs are displayed, analysis adjusted for age, gender, race, diabetes, Hispanic ethnicity, pre‐ and post‐systolic and diastolic blood pressure, interdialytic weight gain, ultrafiltration rate, albumin, Erythropoietin dose, neutrophil to lymphocyte ratio, equilibrated normalized protein catabolic rate, equilibrated Kt/V and body mass index. For full table see Supplemental Material. Note that covariate epo was transformed to square root of epo in all model fits

	Hazard ratio of death (95% CI)
AVF	1.0
AVG	1.12 (0.97‐1.30)
CVC (entire 12 months)	1.55 (1.38‐1.74)
CVC (switched Months 1 to 6)	1.04 (0.97‐1.13)
CVC (switched Months 7 to 12)	1.23 (1.10‐1.38)

AVF = arterio‐venous fistula; AVG = arterio‐venous graft; CVC = central‐venous catheter.

### Sensitivity analysis

A sensitivity analysis for Analysis 1 excluding those that switched multiple times between CVC, AVF, and AVG (N = 2956) was conducted and showed no material differences in the results.

## DISCUSSION

In line with the literature, the current data from a large dialysis provider demonstrate a substantial survival benefit for patients that commence renal replacement therapy with AVF in both univariate and multivariable analysis. *Analysis 1* suggests that early conversion to a non‐CVC access does essentially lead to a survival probability comparable to that of an AVF or AVG access from the start. *Analysis 2* more specifically shows in both univariate and multivariable analyses suggests that once a patient commenced hemodialysis using a CVC, early conversion from CVC to AVG within the first half of the first year on hemodialysis results in a significant survival benefit as compared to remaining on a CVC access (Figure 4). While the same relationship was true for the conversion from CVC to AVF, notably the resulting survival benefit for this conversion remained significant for the entire first year (Figure 4), corroborating a clear advantage of conversion to AVF and to some extent a lower degree of dependency on time compared to AVG.

### Discussion in the light of other studies

These data are consistent with the literature in terms of dialysis accesses at treatment initiation. CVC at dialysis initiation is known to result in an increased risk of death for all patients and subpopulations, associated also in these data with worse outcomes. To the extent studied in this analysis, these data are consistent with previously reported data on change of access and the resulting association with mortality,^1^, ^8^, ^9^, ^10^, ^11^ and showed a survival benefit of changing a CVC access to either AVF or AVG (Figure 3 and Figure 4). Bradbury analyzed data from the first 120 days and reported a significant survival advantage when patients are converted from CVC to either AVF or AVG within this period.^8^ Reduced risk of death was a benefit of conversion within the first 6 months for AVG patients in the currently reported population but a time‐independent benefit for AVF patients. This suggests that if a patient is suitable for conversion to AVF, this always is the preferable approach, however, for patients where vessel quality due to age and/or comorbidities does not allow for AVF placement and maturation, an immediate change in strategy favoring AVG placement may be considered a comparably suitable approach.^14^ Particularly in older patients this evaluation becomes of concern in the light of data from the Monitoring Dialysis Outcomes (MONDO) initiative that has shown that converting from a CVC to either AVF or AVG does provide survival benefits also in elderly patients. Further, and this is of importance when deciding on the appropriate choice of access, the probability of not being converted is highest for elderly females, those elderly patients with cardiovascular disease and those with poor nutritional status.^10^ Given these data one may think critically of the best approach for the individual patient allowing to minimize CVC exposure days that would be putting particularly the elderly patient at elevated risk. Not only is this true on the level of the individual patient's well‐being, but in those prone to a lower life expectancy also on an economic level, where the placement of AVF chosen over that of AVG (considering substantially longer maturation time) to use the access for dialysis may also not be the most cost‐effective choice.^15^ We believe that based on these data, patients will have a substantial beneficial effect on their risk of death if an AVG is placed within the first 6 months. In this context, the importance of predialysis preparation for dialysis (including preparation of a suitable access) gains importance and will result in the lowest risk of death following treatment initiation. Further one could also hypothesize a subsequent reduction in the risk of infection‐related hospitalizations caused by CVC as the access and potentially a decreased need for repeated surgical interventions without the insecurity about successful maturation of the AVF (a period lasting a median time of around 112 days^16^). For the patient requiring an urgent start into dialysis however, the time window of 112 days until an AVF has matured to a point where it can be successfully used for treatment is of importance to plan access management strategies. Carefully selecting those that are more likely to be successfully treated using AVG needs to be a priority and should be done in a multidisciplinary approach involving internist, nephrologist, and the vascular surgeon.

### Strength and limitations

#### *Strengths*

The strengths of this analysis are the large sample size, the wide geographical distribution within the United States, and the consequent generalizability that renders the results generalizable even in the light of the substantial variability of local practice patterns.

#### *Limitations*

The retrospective nature with the risk of bias, confounding requires consideration in combination with the problem of missing data (particularly the lack of data on comorbidities) that is common in studies of that kind, requires consideration when interpreting the results of this study. Both analyses aimed to account for parameters relevant in the context of this bias by adjustment of this analysis, but acknowledge that, as common with retrospective analyses, residual bias and confounding cannot be excluded. Stratification of patients into groups of dialysis access may further have accentuated a possible collider stratification bias where both exposure as well as outcome may associate with a common condition such as older age, present comorbidities, or similar. This risk of a collider bias is in line with data from Quinn and colleagues that claimed the benefit from AVF is solely attributed to patients regardless of whether AVF was used or not.^17^ This is also corroborated by the formal comparison of the groups (Table 1) where significant differences were found. The differences report in the tables are clearly limited to those data available in this retrospective analysis, thus it will not be possible to fully address the question whether the differences in terms of probability of survival and the association with timing (in particular for AVG) are indeed caused by conversion and not by hidden unaccounted factors which, due to unavailability of the respective data, could not be considered in our analyses. Factors that could be thought of in this context range from pathophysiological aspects (such as frailty and the burden of multiple comorbidities) to logistic aspects (such as distance to access surgeons, socioeconomic status and factors not allowing for early conversion). While only prospective studies with a focused design and targeted data collection will allow to fully explain these dynamics and all associated and contributing factors, we aimed in both analyses to adjust for all possible confounding factors that were at our disposition. Unfortunately, one can however not entirely exclude the presence of particularly elderly and sicker patients in the group with worse outcomes. Alternative approaches such as propensity score‐matching may be consider to further reduce the residual error of the estimates; however, in the light of the large sample size, the consistency of the distinct and pathophysiologically relevant associations in the data even after adjustment for a large number of parameters renders the conclusions as possibly valid within the limitations of a retrospective study. Furthermore, it also needs consideration data with a high level of granularity was available, which allowed analyses on the treatment level and also adjustment for a large number of parameters that were deemed relevant to answer the research question. The retrospective nature of this study does limit interpretation and emphasizes the need of additional research in that area. Furthermore, due to documentation issues, no insight into more specific details, such as the type of CVC, location of the AVF, and location or type of AVG, was possible; however, given the large sample size and the level of external validity to be expected from these results, these are negligible concerns and the data reflect the real‐life practice from a large dialysis provider. Further, consequent to the missing data, it was not possible to investigate access‐related complications and events such as hospitalizations due to infectious causes, analyses that may be considered in future analyses. Lastly it also requires mentioning that inclusion of predialysis data would have greatly improved the analysis and allowed for additional insight into the differences between risk modification by dialysis access and conversion and the case mix in terms of comorbidities and other relevant factors.

## CONCLUSION

Predialysis care needs to be prioritized and every patient should undergo a process of careful preparation once it is decided that dialysis is the therapy of choice. Patients that are started on dialysis with a CVC should undergo a careful evaluation by all involved medical disciplines to determine if AVF or AVG is the access of choice. Particularly for the elderly and those with multiple, severe comorbidities, this evaluation and decision is of utmost importance given the median time of first use of 112 days following placement and the survival benefit these data indicate once patients are being converted from a CVC to AVG.

## Supporting information


**Supplemental Figure 1**

**Supplemental Table 1** Hazard ratio of death estimates from Cox regression analysis for 22146 patients who either stayed with CVC, AVF, or AVG and CVC patients that switched either during the first or the second half of the first year to a non‐ CVC access. Full Table of Table 2 in the Main manuscript.

**Supplemental Table 2** Full Estimates for Cox regression models with varying time coefficient for CVC to AVG (N = 4728) and AVF (N = 11843).

## References

[1] Allon M , Daugirdas J , Depner TA , Greene T , Ornt D , Schwab SJ . Effect of change in vascular access on patient mortality in hemodialysis patients. Am J Kidney Dis. 2006;47:469–477. 16490626 10.1053/j.ajkd.2005.11.023

[2] Dhingra RK , Young EW , Hulbert‐Shearon TE , Leavey SF , Port FK . Type of vascular access and mortality in U.S. hemodialysis patients. Kidney Int. 2001;60:1443–1451. 11576358 10.1046/j.1523-1755.2001.00947.x

[3] Pastan S , Soucie JM , McClellan WM . Vascular access and increased risk of death among hemodialysis patients. Kidney Int. 2002;62:620–626. 12110026 10.1046/j.1523-1755.2002.00460.x

[4] Xue JL , Dahl D , Ebben JP , Collins AJ . The association of initial hemodialysis access type with mortality outcomes in elderly Medicare ESRD patients. Am J Kidney Dis. 2003;42:1013–1019. 14582045 10.1016/j.ajkd.2003.07.004

[5] United States Renal Data System. 2016 USRDS annual data report: Epidemiology of kidney disease in the United States. Bethesda, MD: National Institutes of Health, National Institute of Diabetes and Digestive and Kidney Diseases, 2016.

[6] Lacson E Jr , Wang W , Lazarus JM , Hakim RM . Change in vascular access and hospitalization risk in long‐term hemodialysis patients. Clin J Am Soc Nephrol. 2010;5:1996–2003. 20884778 10.2215/CJN.08961209 PMC3001775

[7] Lin H , Zhang R , Xu W , Wang Y . Estimating time‐varying treatment switching effects via local linear smoothing and quasi‐likelihood. Comput Stat Data Anal. 2017;110:50–63.

[8] Bradbury BD , Chen F , Furniss A , et al. Conversion of vascular access type among incident hemodialysis patients: Description and association with mortality. Am J Kidney Dis. 2009;53:804–814. 19268411 10.1053/j.ajkd.2008.11.031

[9] Lacson E Jr , Wang W , Lazarus JM , Hakim RM . Change in vascular access and mortality in maintenance hemodialysis patients. Am J Kidney Dis. 2009;54:912–921. 19748717 10.1053/j.ajkd.2009.07.008

[10] Raimann JG , Barth C , Usvyat LA , et al. Dialysis access as an area of improvement in elderly incident hemodialysis patients: Results from a cohort study from the international monitoring dialysis outcomes initiative. Am J Nephrol. 2017;45:486–496. 28514783 10.1159/000476003

[11] DeSilva RN , Sandhu GS , Garg J , Goldfarb‐Rumyantzev AS . Association between initial type of hemodialysis access used in the elderly and mortality. Hemodial Int. 2012;16:233–241. 22487417 10.1111/j.1542-4758.2011.00661.x

[12] von Elm E , Altman DG , Egger M , Pocock SJ , Gøtzsche PC , Vandenbroucke JP . The strengthening the reporting of observational studies in epidemiology (STROBE) statement: Guidelines for reporting observational studies. Lancet. 2007;370:1453–1457. 18064739 10.1016/S0140-6736(07)61602-X

[13] R Development Core Team . R: A Language and Environment for Statistical Computing. Vienna, Austria: R Foundation for Statistical Computing, 2010.

[14] Disbrow DE , Cull DL , Carsten CG 3rd , Yang SK , Johnson BL , Keahey GP . Comparison of arteriovenous fistulas and arteriovenous grafts in patients with favorable vascular anatomy and equivalent access to health care: Is a reappraisal of the fistula first initiative indicated? J Am Coll Surg. 2013;216:679–685. discussion 85‐6. 23395157 10.1016/j.jamcollsurg.2012.12.021

[15] Hall RK , Myers ER , Rosas SE , O'Hare AM , Colon‐Emeric CS . Choice of hemodialysis access in older adults: A cost‐effectiveness analysis. Clin J Am Soc Nephrol. 2017;12:947–954. 28522655 10.2215/CJN.11631116 PMC5460715

[16] Pisoni RL , Zepel L , Fluck R , et al. International differences in the location and use of arteriovenous accesses created for hemodialysis: Results from the dialysis outcomes and practice patterns study (DOPPS). Am J Kidney Dis. 2018;71:469–478. 29198387 10.1053/j.ajkd.2017.09.012

[17] Quinn RR , Oliver MJ , Devoe D , et al. The effect of predialysis fistula attempt on risk of all‐cause and access‐related death. J Am Soc Nephrol. 2017;28:613–620. 28143967 10.1681/ASN.2016020151 PMC5280018

