# Supplementary figures and images for: Delayed conversion from central venous catheter to non‐catheter hemodialysis access associates with an increased risk of death: A retrospective cohort study based on data from a large dialysis provider
Source: Hemodial Int. 2020 Mar 5;24(3):299–308. doi: 10.1111/hdi.12831 (PMC7496403; doi:10.1111/hdi.12831)

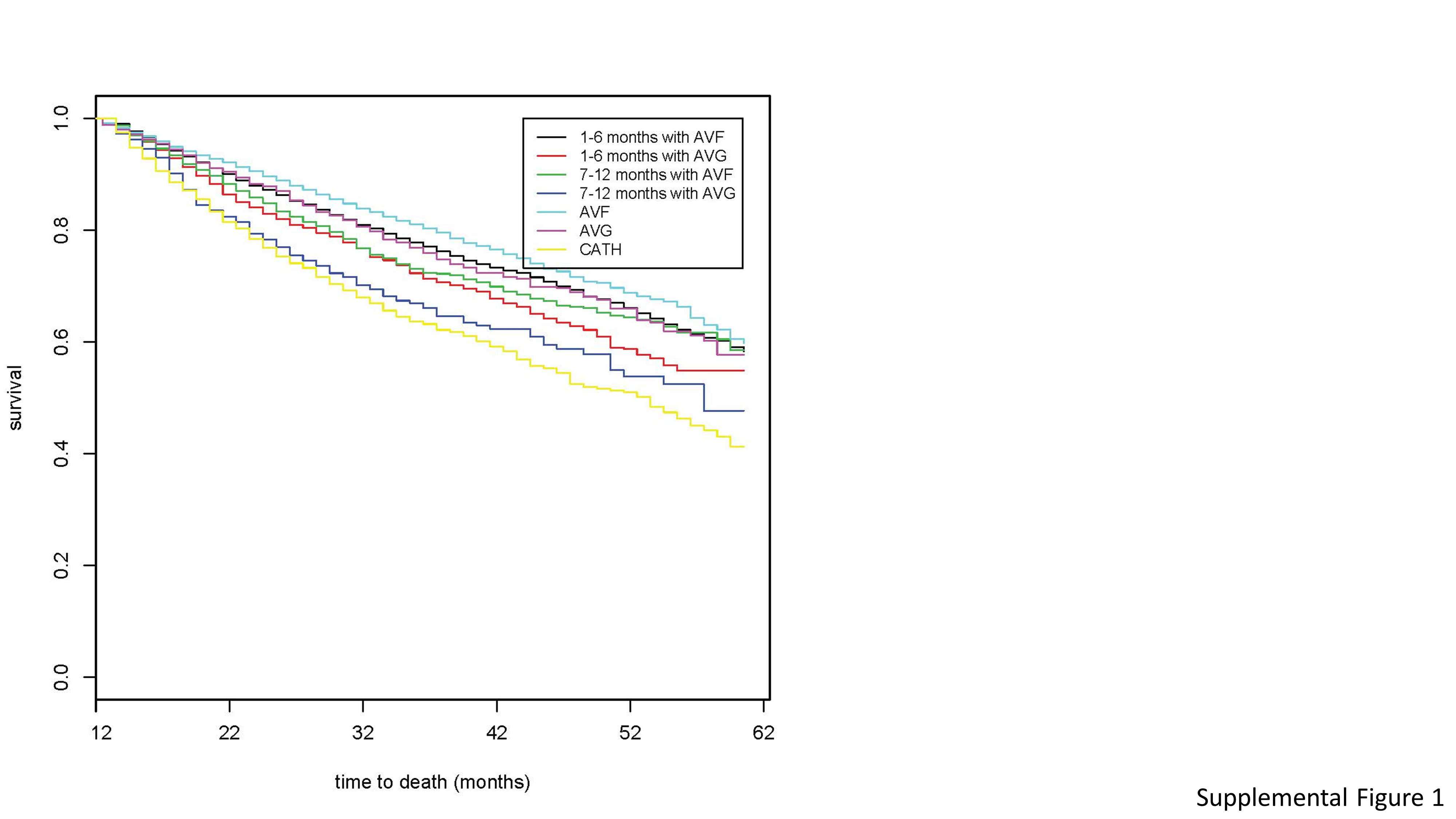

Supplement: Supplementary file 1 — Supplemental Figure 1 [file HDI-24-299-s001.TIF]
