# Supplementary material for: Delayed conversion from central venous catheter to non‐catheter hemodialysis access associates with an increased risk of death: A retrospective cohort study based on data from a large dialysis provider
Source: Hemodial Int. 2020 Mar 5;24(3):299–308. doi: 10.1111/hdi.12831 (PMC7496403; doi:10.1111/hdi.12831)
Supplement: Supplementary file 2 — Supplemental Table 1 Hazard ratio of death estimates from Cox regression analysis for 22146 patients who either stayed with CVC, AVF, or AVG and CVC patients that switched either during the first or the second half of the first year to a non‐ CVC access. Full Table of Table 2 in the Main manuscript. [file HDI-24-299-s002.docx]

**Supplemental Table 1:** Hazard ratio of death estimates from Cox regression analysis for 22146 patients who either stayed with CVC, AVF, or AVG and CVC patients that switched either during the first or the second half of the first year to a non- CVC access. Full Table of **Table 2** in the Main manuscript.

|  | Hazard ratio of death (95% CI) |
| --- | --- |
| AVF | 1.0 |
| AVG | 1.12 (0.97 to 1.30) |
| CVC (entire 12 months ) | 1.55 (1.38 to 1.74) |
| CVC (switched Months 1 to 6) | 1.04 (0.97 to 1.13) |
| CVC (switched Months 7 to 12) | 1.23 (1.10 to 1.38) |
| Age [per 1 year] | 1.03 (1.03 to 1.04) |
| White race [yes/no] | 1.55 (1.44 to 1.68) |
| Male gender [yes/no] | 1.22 (1.13 to 1.31) |
| Diabetic [yes/no] | 1.18 (1.09 to 1.27) |
| Hispanic ethnicity [yes/no] | 0.70 (0.63 to 0.77) |
| Pre HD SBP [per 1 mmHg] | 0.98 (0.97 to 0.98) |
| Pre HD DBP [per 1 mmHg] | 1.03 (1.02 to 1.04) |
| Post HD SBP [per 1 mmHg] | 1.02 (1.01 to 1.03) |
| Post HD DBP [per 1 mmHg] | 0.97 (0.95 to 0.98) |
| IDWG [per 1 % body weight] | 0.94 (0.85 to 1.03) |
| Ultrafiltration rate [per 1 mL/hr/kg body weight] | 1.09 (1.06 to 1.13) |
| Albumin [per 1 g/dL] | 0.42 (0.38 to 0.46) |
| Square root of EPO [per 1 unit] | 1.00 (1.00 to 1.01) |
| NLR [per 1 unit] | 1.07 (1.06 to 1.08) |
| enPCR [per 1 g/kg/d] | 0.40 (0.34 to 0.49) |
| eKt/V [per 1 unit] | 0.91 (0.80 to 1.04) |
| BMI [per 1 kg/m^2^] | 0.99 (0..99 to 1.00) |

Abbreviations: arterio-venous fistula (AVF), arterio-venous graft (AVG), central-venous catheter (CVC), hemodialysis (HD), systolic blood pressure (SBP), diastolic blood pressure (DBP), interdialytic weight gain (IDWG), erythropoietin dose (EPO), neutrophil-lymphocyte ratio (NLR), equilibrated normalized protein catabolic rate (enPCR), body mass index (BMI).
