# Supplementary material for: Delayed conversion from central venous catheter to non‐catheter hemodialysis access associates with an increased risk of death: A retrospective cohort study based on data from a large dialysis provider
Source: Hemodial Int. 2020 Mar 5;24(3):299–308. doi: 10.1111/hdi.12831 (PMC7496403; doi:10.1111/hdi.12831)
Supplement: Supplementary file 3 — Supplemental Table 2 Full Estimates for Cox regression models with varying time coefficient for CVC to AVG (N = 4728) and AVF (N = 11843). [file HDI-24-299-s003.docx]

**Supplemental Table 2:** Full Estimates for Cox regression models with varying time coefficient for CVC to AVG (N=4728) and AVF (N=11843).

|  | CVC to AVF  Hazard ratio of death (95% CI) | CVC to AVG  Hazard ratio of death (95% CI) |
| --- | --- | --- |
| Age [per 1 year] | 1.03 (1.03 to 1.04) | 1.03 (1.02 to 1.04) |
| White race [yes/no] | 1.33 (1.18 to 1.50) | 1.33 (1.14 to 1.55) |
| Male gender [yes/no] | 1.05 (0.94 to 1.17) | 1.15 (1.00 to 1.32) |
| Diabetic [yes/no] | 1.00 (0.90 to 1.12) | 0.99 (0.87 to 1.13) |
| Hispanic ethnicity [yes/no] | 0.69 (0.58 to 0.81) | 0.78 (0.64 to 0.96) |
| Pre HD SBP [per 1 mmHg] | 0.99 (0.98 to 0.99) | 0.99 (0.98 to 0.99) |
| Pre HD DBP [per 1 mmHg] | 1.01 (1.00 to 1.02) | 1.01 (1.00 to 1.02) |
| Post HD SBP [per 1 mmHg] | 1.00 (0.99 to 1.00) | 1.00 (0.99 to 1.01) |
| Post HD DBP [per 1 mmHg] | 0.99 (0.98 to 1.00) | 0.99 (0.97 to 1.00) |
| IDWG [per 1 % body weight] | 1.10 (1.04 to 1.16) | 1.03 (0.97 to 1.10) |
| Ultrafiltration rate [per 1 mL/hr/kg body weight] | 1.02 (1.00 to 1.05) | 1.03 (1.00 to 1.05) |
| Albumin [per 1 g/dL] | 0.62 (0.57 to 0.69) | 0.68 (0.60 to 0.76) |
| NLR [per 1 unit] | 1.02 (1.01 to 1.03) | 1.02 (1.01 to 1.03) |
| enPCR [per 1 g/kg/d] | 0.70 (0.55 to 0.91) | 0.74 (0.54 to 1.02) |
| eKt/V [per 1 unit] | 0.93 (0.81 to 1.06) | 0.82 (0.69 to 0.98) |
| BMI [per 1 kg/m^2^] | 1.00 (1.00 to 1.01) | 1.0 (1.00 to 1.01) |
| Square root of EPO [per 1 unit] | 1.00 (1.00 to 1.00) | 1.0 (1.00 to 1.00) |

Abbreviations: arterio-venous fistula (AVF), arterio-venous graft (AVG), central-venous catheter (CVC), hemodialysis (HD), systolic blood pressure (SBP), diastolic blood pressure (DBP), interdialytic weight gain (IDWG), erythropoietin dose (EPO), neutrophil-lymphocyte ratio (NLR), equilibrated normalized protein catabolic rate (enPCR), body mass index (BMI).
